# Analgesic and Antiinflammatory Activities of the Aqueous Extract from *Plectranthus amboinicus* (Lour.) Spreng. Both *In Vitro* and *In Vivo*

**DOI:** 10.1155/2012/508137

**Published:** 2011-09-11

**Authors:** Yung-Jia Chiu, Tai-Hung Huang, Chuan-Sung Chiu, Tsung-Chun Lu, Ya-Wen Chen, Wen-Huang Peng, Chiu-Yuan Chen

**Affiliations:** ^1^School of Chinese Pharmaceutical Sciences and Chinese Medicine Resources, College of Pharmacy, China Medical University, no. 91 Hsueh-Shih Road, Taichung 40402, Taiwan; ^2^School of Pharmacy, College of Pharmacy, China Medical University, no. 91 Hsueh-Shih Road, Taichung 40402, Taiwan; ^3^Hsin Sheng College of Medical Care and Management, no. 418 Zhongfeng Road, Taoyuan 32544, Taiwan; ^4^Institute of Infectious Diseases and Vaccinology, National Health Research Institutes, no. 35 Keyan Road, Miaoli County 35053, Taiwan; ^5^Graduate Program in Life Science, University of Maryland, Baltimore, MD 21201, USA; ^6^Greenebaum Cancer Center, University of Maryland, Baltimore, MD 21201, USA; ^7^Graduate Institute of Natural Healing Sciences, Nanhua University, no. 55, Section 1, Nanhua Road, Zhongkeng, Dalin Township, Chiayi County 62248, Taiwan

## Abstract

*Plectranthus amboinicus* (Lour.) Spreng. is a native Labiatae plant of Taiwan. The plants are commonly used in Chinese folk medicine for the treatment of cough, fever, sore throats, mumps, and mosquito bite. The aim of this study was to investigate the analgesic and antiinflammatory properties of the aqueous extract from *Plectranthus amboinicus* (PA) *in vivo* and *in vitro*. PA inhibited pain induced by acetic acid and formalin, and inflammation induced by carrageenan. The anti-inflammatory effect of PA was related to modulating antioxidant enzymes' activities in the liver and decreasing the Malondialdehyde (MDA) level and the production of tumor necrosis factor alpha (TNF-**α**), and cyclooxygenase2 (COX-2) in edema-paw tissue in mice. *In vitro* studies show that PA inhibited the proinflammatory mediators in RAW 264.7 cells stimulated with lipopolysaccharide (LPS). PA blocked the degradation of I**κ**B-**α** and nuclear translocation of NF-**κ**B p65 subunit. Finally, the amount of carvacrol in the aqueous extract of PA was 1.88 mg/g extract. Our findings suggest that PA has analgesic and anti-inflammatory activities. These effects were mediated by inhibiting the proinflammatory mediators through blocking NF-**κ**B activation. Meanwhile, the effects observed in this study provide evidence for folkloric uses of *Plectranthus amboinicus* (Lour.) Spreng. in relieving pain and inflammation.

## 1. Introduction

Inflammation is the result of host response to tissue injuries or pathogenic challenges and ultimately leads to the restoration of a normal tissue structure and function. Acute inflammation is a limited beneficial process, particularly in response to infectious pathogens, whereas chronic inflammation is an undesirable persistent phenomenon that can lead to the developments of inflammatory diseases [1]. Prolonged inflammation contributes to the pathogenesis of many inflammatory diseases, such as, metabolic disease [2], atherosclerosis [3], obesity, cardiovascular disease [4], rheumatoid arthritis [5], and cancer [6]. 

Acute inflammation, which is typically characterized by redness, swelling, pain, and heat, is one of the most important host defense mechanisms against invading pathogens. Lipopolysaccharide (LPS) from gram-negative bacteria is well known to cause bacterial sepsis mediated through the activation of monocytes, neutrophils, and macrophages [7]. Sometimes the activation of these cells may induce over secretion of various proinflammatory and toxicity mediating molecules such as tumor necrosis factor alpha (TNF-*α*), interleukin-6 (IL-6), eicosanoids, and nitric oxide (NO) [8]. However, excessive inflammatory response has damaging effects, such as septic shock, which can lead to multiple organ dysfunction syndrome and death. Prostaglandins (PGs) and NO are two important proinflammatory mediators and inhibition of productions of both PGs and NO via the inhibition of their synthases, cyclooxygenase2 (COX-2), and inducible nitric oxide synthase (iNOS), respectively, has been demonstrated beneficial in treating inflammatory disease [9]. Antiinflammatory drugs such as steroids or nonsteroidal antiinflammatory drugs (NSAIDs) have a number of adverse side effects, such as gastrointestinal discomfort, inhibition of platelet aggregation, and liver and kidney toxicity [10]. Thus, there is considerable research interest in the identification of new antiinflammatory agents from plants used in traditional medicine.

*Plectranthus amboinicus *(Lour.) Spreng. is a native Labiatae plant of Taiwan. The plants are commonly used in Chinese folk medicine for the treatment of cough, fever, sore-throats, mumps, and mosquito bite [11, 12]. Previous study showed that the ethanol extract of *Plectranthus amboinicus* possesses nephroprotective and antioxidant effects against acetaminophen-induced nephrotoxicity and strong diuretics effect in rats [13]. *Plectranthus amboinicus* also showed the ability to treat collagen-induced arthritis in rats [14]. However, the therapeutic potential of *Plectranthus amboinicus* for inflammatory diseases remains fully unclear. The purpose of this study is to examine the analgesic, antioxidant, and antiinflammatory effects of the aqueous extract of *Plectranthus amboinicus* (PA) in *in vivo* models and the antiinflammatory mechanisms of PA in *in vitro *models. The peripheral analgesic activity of PA was determined by the acetic acid-induced writhing response and formalin test. We also analyzed the levels of the antioxidant enzymes in the liver and several proinflammatory markers in the paw tissue of carrageenan-induced edema models. The antiinflammatory mechanisms of PA were revealed using LPS-induced RAW 264.7 macrophages model. Our results demonstrate that the aqueous extract of *Plectranthus amboinicus* has the analgesic and antiinflammatory abilities and suggest that *Plectranthus amboinicus* has the therapeutic potential to be used as an alternative medicine for inflammatory diseases.

## 2. Materials and Methods

### 2.1. Preparation of Plant Extract

The plants of *Plectranthus amboinicus* were collected in Taichung of Taiwan in July 2008 and were identified by Dr. Chao-Lin Kuo, leader of the School of Chinese Medicine Resources (SCMR). Fresh *Plectranthus amboinicus* (6 kg) was minced using a mixer grinder with 10 L double distilled water at room temperature. The juice was filtered, concentrated, and freeze dried to obtain crude aqueous extract with a yield ratio of 0.954% (w/w, with reference to fresh material). Essential oil of* Plectranthus amboinicus *has been studied for its chemical composition by hydro distillation. Totally 26 compounds were identified by GC and GC-MS. The major chemical compounds were carvacrol followed by thymol, *α*-humulene, undecanal, *γ*-terpinene, *ρ*-cymene, caryophyllene oxide, *α*-terpineol, and *β*-selinene [12]. High performance liquid chromatography (HPLC) method has been developed for the analysis of essential oil [15]. Thus, the aqueous extract of PA was then analyzed with HPLC analyses, and the peaks were identified by comparison with the standard solutions (carvacrol).

### 2.2. Animals

Male ICR mice (18–22 g) were obtained from the animal center of school of medicine in National Taiwan University. Animals used in this study were housed and cared in accordance with the NIH guide for the care and use of laboratory animals. The experimental protocol was approved by the Committee on Animal Research, China Medical University, under the code 2006-14-N. Mice were housed in standard cages at a constant temperature of 22 ± 1°C and relative humidity 55 ± 5% with 12 h light-dark cycle for at least 1 week before the experiments. All tests were conducted under the guidelines of the International Association for the Study of Pain [16]. Each animal experiment was performed in five groups of ten mice each.

### 2.3. Chemicals

Carrageenan, indomethacin, carvacrol, Griess reagent, lipopolysaccharide, 3-(4,5-dimethylthiazol-2-yl)-2,5-diphenyl tetrazolium bromide (MTT), and other chemicals were purchased from Sigma-Aldrich Chemical Co (St. Louis, Mo, USA). Formalin was purchased from Nihon Shiyaku Industry Ltd. (Japan). Murine TNF-*α* enzyme-link immunosorbent assay (ELISA) Development Kit (900-k54) was purchased from PeproTech EC Ltd. Antibodies of iNOS, COX-2, and I*κ*B-*α* were purchased from Abcam. Indomethacin was suspended in 0.5% (w/v) carboxymethylcellulose sodium (CMC) and administered intraperitoneally (i.p.) to animals.

### 2.4. Preliminary Phytochemical Analysis

HPLC analysis was performed on a Synergi Fusion-RP 80, column 4 *μ*m, 250 × 4.6 mm; mobile phases: 0.2% formic acid : methanol (45 : 55, v/v); flow rate: 1 mL/min. Peaks were analyzed spectroscopically at 274 nm with a UV-visible-light detector (SPD-M10AVP, Shimadzu). The PA solutions were quantified by spiking with a known amount of standard (carvacrol) and also by comparing the area under curve. The repeatability of the method was evaluated by injecting the solution of PA and standard solution for three times, and the relative standard deviation (RSD) percentage was calculated.

### 2.5. Acetic Acid-Induced Writhing Test

The writhing test in mice was conducted as described in the previous study [16]. Male ICR mice (ten per group) were fasted for 24 h before the experiment, with free access to water. The writhes were induced by intraperitoneal injection of 1.0% acetic acid in distilled water (0.1 mL/10 g body weight). Preliminary data showed that the dosage in 1.0 g/kg possesses maximum antiinflammatory effects, and then we chose three doses (0.1, 0.5, and 1.0 g/kg) for following animal experiment. Mice were administered orally with PA (0.1, 0.5, and 1.0 g/kg dissolved in distilled water) 60 min prior to chemical induction of writhes and the same volume of distilled water by oral administration as the vehicle control. Indomethacin (10 mg/kg, i.p.) was administered 30 min prior to acetic acid injection. Mice were placed in an observation box separately and the number of writhing responses was counted within 10 min.

### 2.6. Formalin Test

The test was conducted according to the method described in the previous study [17]. Male ICR mice (ten per group) were fasted for 24 h before the experiment, with free access to water. Twenty microliter of 5% formalin in distilled water was then injected subcutaneously into the right hind paw of mice to cause pain. Mice were administered orally with PA (0.1, 0.5, and 1.0 g/kg dissolved in distilled water) 60 min before formalin treatment and the same volume of distilled water by oral administration as the vehicle control. Indomethacin (10 mg/kg, i.p.) was administered 30 min before formalin treatment. These mice were individually placed in a transparent Plexiglas cage (25 × 15 × 15 cm). The time spent licking and biting the injected paw as the index of pain was recorded separately from 0 to 5 min as early phase or neurogenic pain and from 20 to 30 min as late phase or inflammatory pain [18].

### 2.7. Carrageenan-Induced Mice Paw Edema

This method was previously described and was used with some modifications [19]. Male ICR mice (ten per group) were fasted for 24 h before the experiment with free access to water. The mice were injected subcutaneously with 50 *μ*L of 1% carrageenan solution in normal saline (0.9% w/v NaCl) into the subplantar region of the right hind paw. PA (0.1, 0.5, and 1.0 g/kg, p.o.) was administered at 120 min after carrageenan injection and the same volume of distilled water by oral administration as the vehicle control. Indomethacin (10 mg/kg, i.p.) was administered at 150 min after carrageenan injection. The paw volume was measured before (0 h) and at intervals of 1, 2, 3, and 4 h after carrageenan injection using a plethysmometer.

For the malondialdehyde (MDA), TNF-*α*, and COX-2 assays, the whole right hind paws were collected at the third hour after carrageenan injection. The right hind paw tissue was rinsed in ice-cold normal saline and immediately placed in cold normal saline four times their volume and finally homogenized at 4°C. Then, the homogenate was centrifuged at 12,000 rpm for 5 min. The supernatant was obtained and stored at −80°C for the MDA, TNF-*α*, and COX-2 assays.

For the antioxidant enzyme activity assays, liver tissues were collected at the third hour after carrageenan injection and rinsed in ice-cold normal saline and immediately placed in cold normal saline of the same volume and finally homogenized at 4°C. Then, the homogenate was centrifuged at 12,000 rpm for 5 min. The supernatant was obtained and stored at −80°C for the antioxidant enzyme (superoxide dismutase, glutathione peroxidase, and glutathione reductase) activity assays.

### 2.8. Malondialdehyde Assay

Malondialdehyde (MDA) was evaluated by the thiobarbituric acid-reacting substance (TBARS) method [20]. Briefly, MDA can react with thiobarbituric acid (TBA) under the acidic and high-temperature conditions. MDA and TBA then formed a red-complex TBARS, which can be measured colorimetrically. The absorbance of TBARS was determined by measurement of 532 nm.

### 2.9. Antioxidant Enzymes' Activities

Liver tissue homogenates were collected for the estimation of superoxide dismutase (SOD), glutathione peroxidase (GPx) and glutathione reductase (GRx) enzyme to detect the antioxidant activities of PA [21].

### 2.10. Tissue COX-2 by Quartz Crystal Microbalance Assay

The P-sensor 2000 designed by ANT (Asia New Technology, Taiwan) is based on the principle of piezoelectric biosensor. P-sensor 2000 based on quartz crystal microbalance (QCM) was used to monitor the antibody-antigen interaction in real time. It is made of three portions including electronic oscillation circuit, frequency counter, and piezoelectric quartz of fixed biosensor molecule (p-chip). The piezoelectric quartz crystal consists of a quartz crystal slab with a layer of gold electrode on each side. It is the signal conversion component of the piezoelectric sensor chip and can convert the result sensed by the sensor molecule into electronic signal to be amplified. The function of gold electrodes is mainly to introduce an oscillating electric field perpendicular to the surface of the chip so that the internal part of the chip generates mechanical oscillation because of the piezoelectric effect. If the thickness of the quartz crystal is fixed, the mechanical oscillation will be generated at a fixed frequency. Using a suitable electronic oscillation circuit, the resonant frequency can be measured. The PBS, a mobile carrier, would flow through the sensor cell with the antibody-immobilized chip in flow rate of 30 *μ*L/min and clean the fluid lines of QCM, alternating with the 1 N NaOH and 1 N HCl solution and ultra-pure water before the measurement. After the introduction of PBS to fill the sensor cell, the frequency shift of QCM reached a steady equilibrium (“*F*” < 0.2 Hz/min) and was defined as a zero baseline, “*F*_0_”. Upon the injection of supernatant solution into the sensor cell, the dynamic interactions between antigens and immobilized antibodies were monitored, and the frequency shifts were recorded for the next steady equilibrium, “*F*”. Thus, the apparent frequency change of crystal oscillator, “*F*”, can be measured by subtracting “*F*” from “*F*_0_”. All of PBS and diluted sera solutions were filtered with Millex-GP filter unit (0.22 *μ*m, PES membrane; Millipore, Ireland) and degassed before used. The sensor chips were disposable to ensure the sensitivity and reproducibility of each of the QCM experiments. With a temperature controller, the temperature of the sensor cell was controlled at constant temperature of 25°C to suppress the fluctuations of kinetics by ambient environment.

### 2.11. Tissue and Cells Release TNF-*α* by ELISA

TNF-*α* level was determined using a commercially available enzyme-linked immunosorbent assay (ELISA) kit according to the manufacturer's instruction. The absorbance at 450 and 540 nm was measured on a microplate reader (VersaMax, Mass, USA).

### 2.12. Cell Culture and Cell Viability

RAW 264.7 macrophage cell line was obtained from Culture Collection and Research Center (Hsinchu, Taiwan). Cells were grown at 37°C in Dulbecco's modified Eagle's medium supplemented with 10% FBS, penicillin (100 units/mL), and streptomycin sulfate (100 *μ*g/mL) in a humidified 5% CO_2_ atmosphere. Cell viability was assessed by the mitochondrial-dependent reduction of MTT to purple formazan. Cells were incubated with MTT (0.5%) for 4 h at 37°C. The medium was removed by aspiration, and formazan crystals were dissolved in DMSO. The absorbance was measured at 550 nm on a microplate reader, and % survival was determined by comparison with control group.

### 2.13. Nitrite Measurement

The nitrite concentration in the medium was measured according to the Griess reaction, and the calculated concentration was taken as an indicator of NO production. The supernatant of cell cultures was mixed with an equal volume of Griess reagent (1% sulfanilamide in 5% phosphoric acid and 0.1% naphthyl ethylenediamine dihydrochloride in water). The optical density at 550 nm was measured and calculated against a sodium nitrite standard curve.

### 2.14. Western Blot Analysis

Cells were lysed at 4°C in RIPA buffer containing 50 mM Tris-HCl (pH 7.4), 150 mM NaCl, 1% Triton X-100, 0.25% Sodium deoxycholate, 5 mM EDTA (pH 8.0), and 1 mM EGTA and supplemented with protease and phosphatase inhibitors. After 20 min of lysis on ice, cell debris was removed by microcentrifugation, followed by quick freezing of the supernatants. The protein concentration was determined by the Bradford method. Equal amounts of proteins were separated onto SDS-polyacrylamide gels and then electrophoretically transferred from the gel onto a PVDF membrane (Millipore, Bedford, Mass, USA). After blocking, the membrane was reacted with specific primary antibodies overnight at 4°C and then incubated with horseradish peroxidase conjugated secondary antibody for 1 h. The blots were visualized using the ECL-Plus detection kit (PerkinElmer Life Sciences, Inc. Boston, Mass, USA).

### 2.15. Immunofluorescence

Cells were fixed with 2% paraformaldehyde for 20 min. The cells were incubated with 0.1% Triton X-100 for 30 min then blocked with 1% BSA for 30 min. Cells were probed with mouse anti-p65 antibody (Santa Cruz Biochemicals, Santa Cruz, Calif, USA, 1 : 500) overnight at 4°C, followed by FITC-conjugated goat antimouse IgG antibody (Sigma, St. Louis, Mo, USA, diluted 1 : 200) 1 h at 37°C, washed with PBS three times and then stained with DAPI for 10 min. NF-*κ*B p65 subunit was observed with a laser scanning confocal microscope.

### 2.16. Statistical Analysis

All the data were expressed as mean  ±  SEM. Statistical analysis was carried out using one-way ANOVA, followed by Scheffe's multiple range tests. The criterion for statistical significance was *P *< 0.05.

## 3. Results

### 3.1. Phytochemical Study of PA

**Figure 1:**
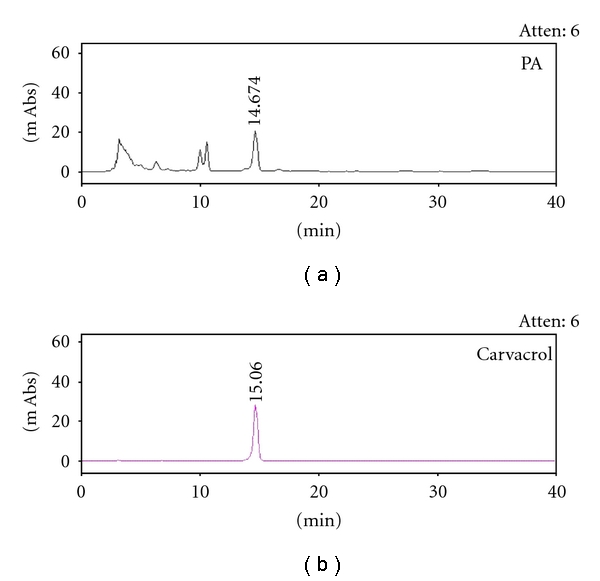
Representative HPLC chromatograms of carvacrol (*R_t_* = 15.060) and PA extract.

Phytochemical study of aqueous extracts from PA revealed the abundant presence of carvacrol. The content and variety of carvacrol which has a maximum absorbance at 274 nm is 1.88 ± 0.53 mg/g extract (Figure 1).

### 3.2. Analgesic Effect of PA in Mice

**Figure 2:**
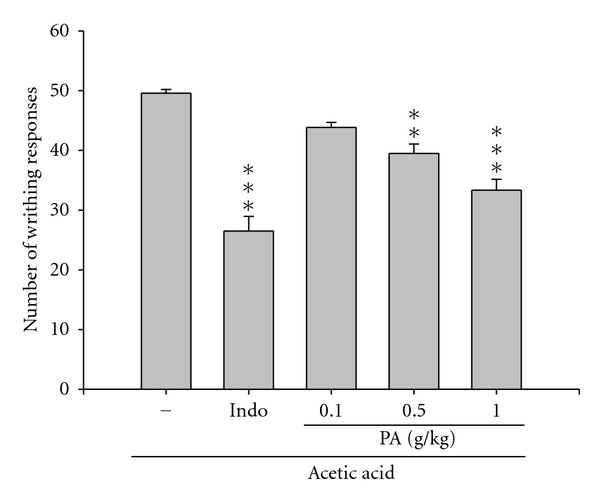
Analgesic effect of PA on acetic acid-induced writhing response in mice. Indomethacin (Indo 10 mg/kg) was used as a therapeutic control. The number of muscular contractions was evaluated as described in Section 2. Treatment of PA at 0.5 and 1.0 g/kg and indomethacin at 10 mg/kg showed inhibition of writhing number compared to the control. Each value represents as mean ± SEM (*n* = 10). **P < 0.01, ***P < 0.001 as compared with the acetic acid-treated only group.

**Figure 3:**
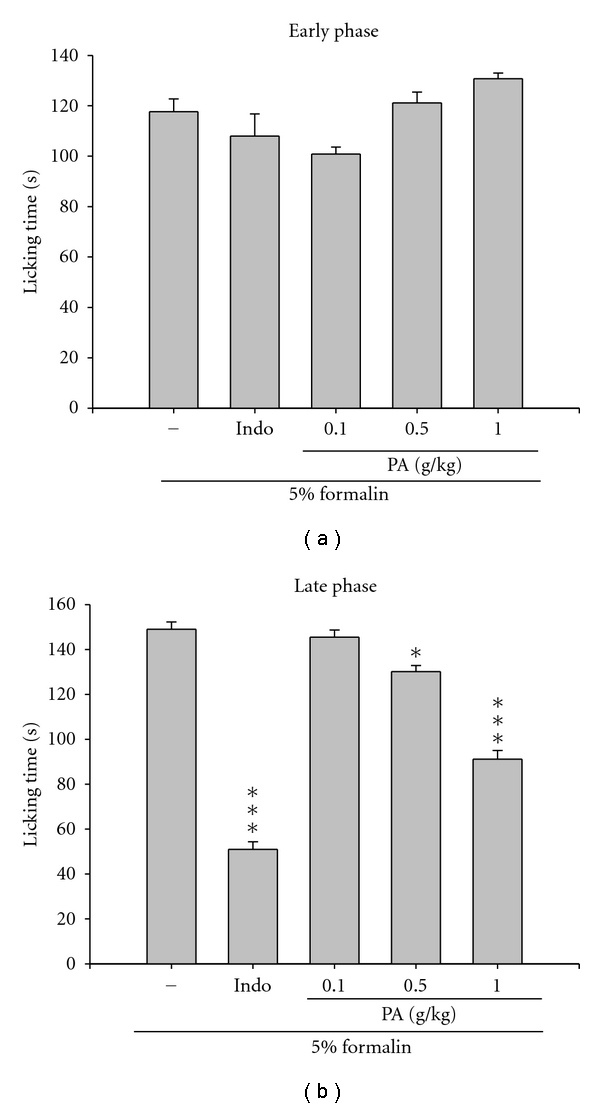
Effects of PA on the (a) early phase and (b) late phase in formalin test in mice. The index of pain (early phase and late phase) was evaluated as described in Section 2. PA showed a dose-dependent effect on the decrease of licking time in the late phase of formalin-induced pain. Each value represents as mean ± SEM (*n* = 10). *P < 0.05, ***P < 0.001 as compared with the formalin-treated only group.

Effect of the PA in decreasing the acetic acid-induced writhing responses in mice which indicates that the analgesic activity is presented in Figure 2. Treatment of PA at 0.5 and 1.0 g/kg and indomethacin at 10 mg/kg showed the inhibition of writhing number compared to the control (*P *< 0.01–0.001). Moreover, PA also showed a dose-dependent effect on the decrease of licking time in the late phase of formalin-induced pain (Figure 3(b), *P *< 0.05–0.001) though there are no significant inhibitions in the early phase (Figure 3(a)).

### 3.3. PA-Inhibited Carrageenan-Induced Edema and Inflammation in Mice Paw Tissue

**Figure 4:**
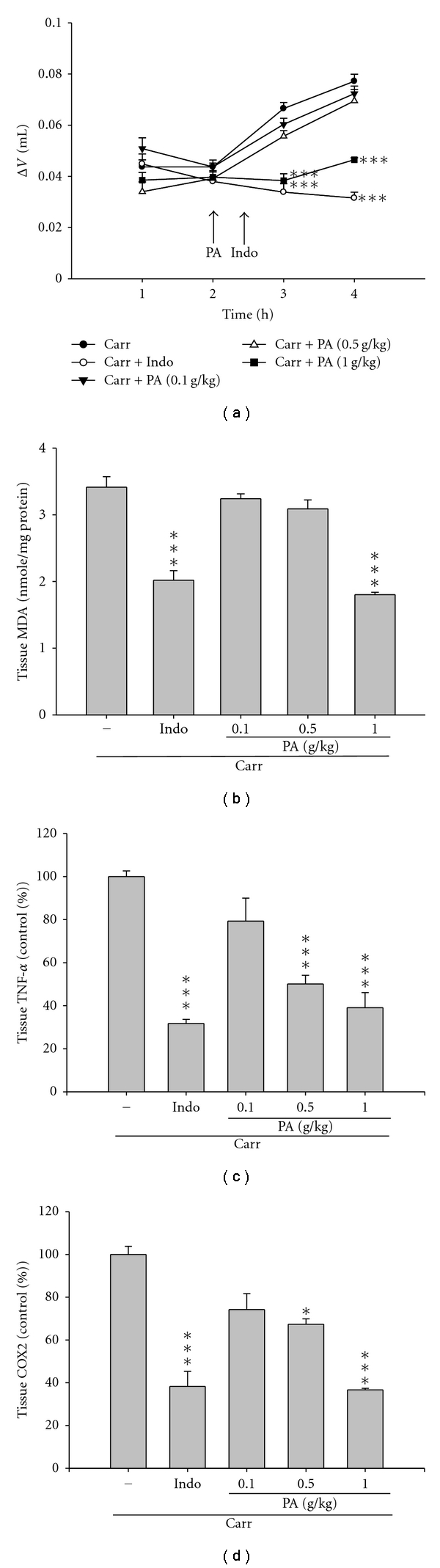
Inhibitory effects of PA on carrageenan-induced mice paw edema and inflammation. (a) Treatment of PA (1.0 g/kg) significantly reduced the paw edema formation. Delta volume (Δ *V*) represents the degree of swelling of carrageenan-treated paw. (b) Treatment of PA (1.0 g/kg) significantly decreased the levels of MDA, TNF-*α*, and COX-2. MDA concentration and the levels of TNF-*α* and COX-2, showing as percentage, were presented as mean ± SEM (*n* = 10). *P < 0.05, ***P < 0.001 as compared with the carrageenan-treated only group.

The carrageenan-induced mice paw edema is a biphasic process [22]. In the early hyperemia, 0–2 h after carrageenan injection, there is a release of histamine, serotonin, and bradykinin to increase vascular permeability. The inflammatory edema reached its maximum level at the third hour and after that it started declining. In our study, the paw edema was increased and reached maximally at 4 h after carrageenan injection. Treatment of PA (1.0 g/kg) significantly reduced the paw edema formation (*P *< 0.001) as shown in Figure 4(a). The inhibition rate at 4 h was shown as 41.2% and 62.3% with the treatment of PA (1.0 g/kg) and indomethacin, respectively. 

Previous reports have demonstrated that accumulations of MDA, TNF-*α*, and COX-2 are indications of inflammation. Thus, we set out to measure the MDA level using TBARS method, the TNF-*α* level using ELISA, and the COX-2 level using QCM in paw tissues from carrageenan-induced edema model mice. As expected, the levels of MDA, TNF-*α*, and COX-2 were increased in the carrageenan-induced paw edema mice (Figures 4(b), 4(c), and 4(d)). However, treatment of PA (1.0 g/kg) significantly decreased the levels of MDA, TNF-*α*, and COX-2 (*P *< 0.001, Figures 4(b), 4(c), and 4(d), resp.). Moreover, treatment of PA at lower dose (0.5 g/kg) also decreased the levels of TNF-*α* and COX-2 (*P  *<  0.001 and 0.05, resp.).

### 3.4. Antioxidant Abilities of PA in Mice

**Figure 5:**
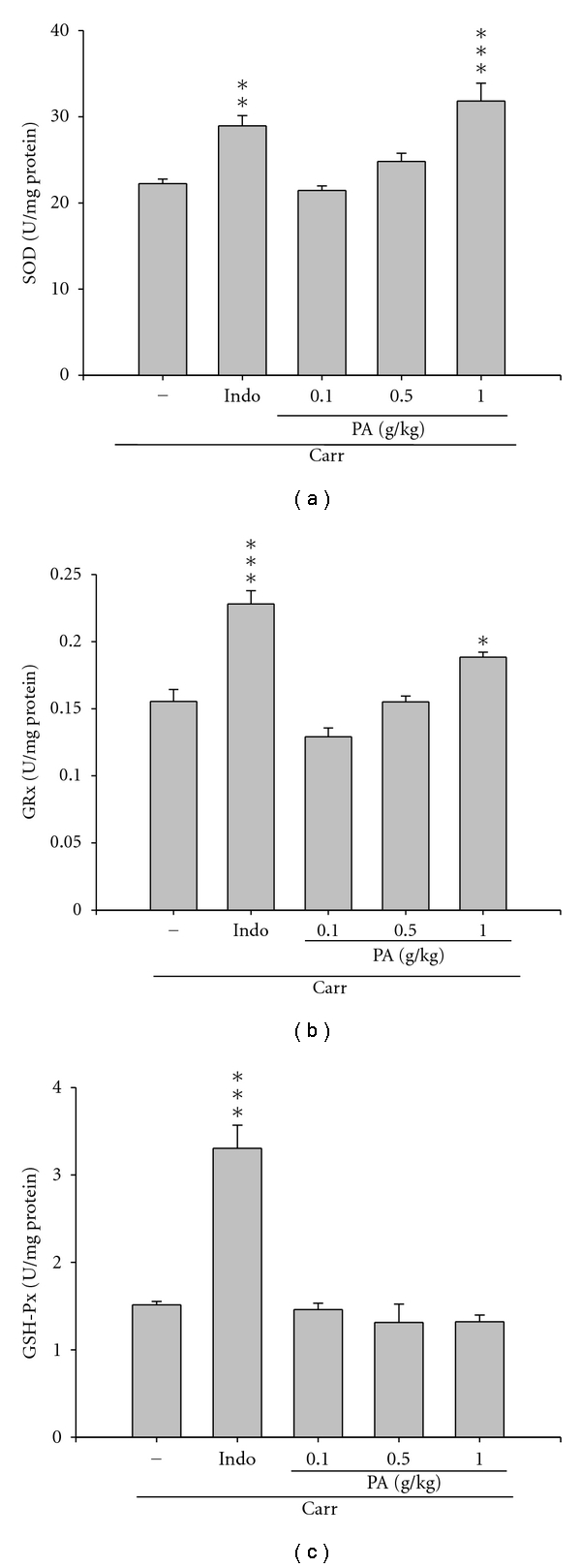
Antioxidant abilities of PA on carrageenan-induced mice. Liver tissues from carrageenan-treated mice were used to analyze the activities of superoxide dismutase (SOD), glutathione reductase (GRx), and glutathione peroxidase (GPx). (a) SOD activity increased significantly after treatment with indomethacin and PA (1.0 g/kg). (b) GRx activities in the liver tissues increased significantly with the treatment of indomethacin and PA (1.0 g/kg). (c) There are no significant inhibitions of GPx activities when treatment with any dose of PA, which was comparable to the treatment of indomethacin. All values represent as means ± SEM (*n* = 10). *P < 0.05, ***P < 0.001 as compared with the carrageenan-treated only group.

To investigate the antioxidant abilities of PA, the activities of antioxidant enzymes (SOD, GPx, and GRx) at the third hour after carrageenan injection were investigated. In our result, SOD activity increased significantly after treatment with indomethacin and PA (1.0 g/kg) (*P *< 0.01 and *P *< 0.001, resp.) (Figure 5(a)). GRx activities in the liver tissues increased significantly with the treatment of indomethacin and PA (1.0 g/kg) (*P *< 0.001 and *P *< 0.05, resp.) (Figure 5(b)). There are no significant inhibitions of GPx activities when treatment with any dose of PA, which was comparable to the treatment of indomethacin (*P *< 0.001) (Figure 5(c)).

### 3.5. PA-Inhibited LPS-Induced TNF-*α* and NO Production in RAW 264.7 Cells

**Figure 6:**
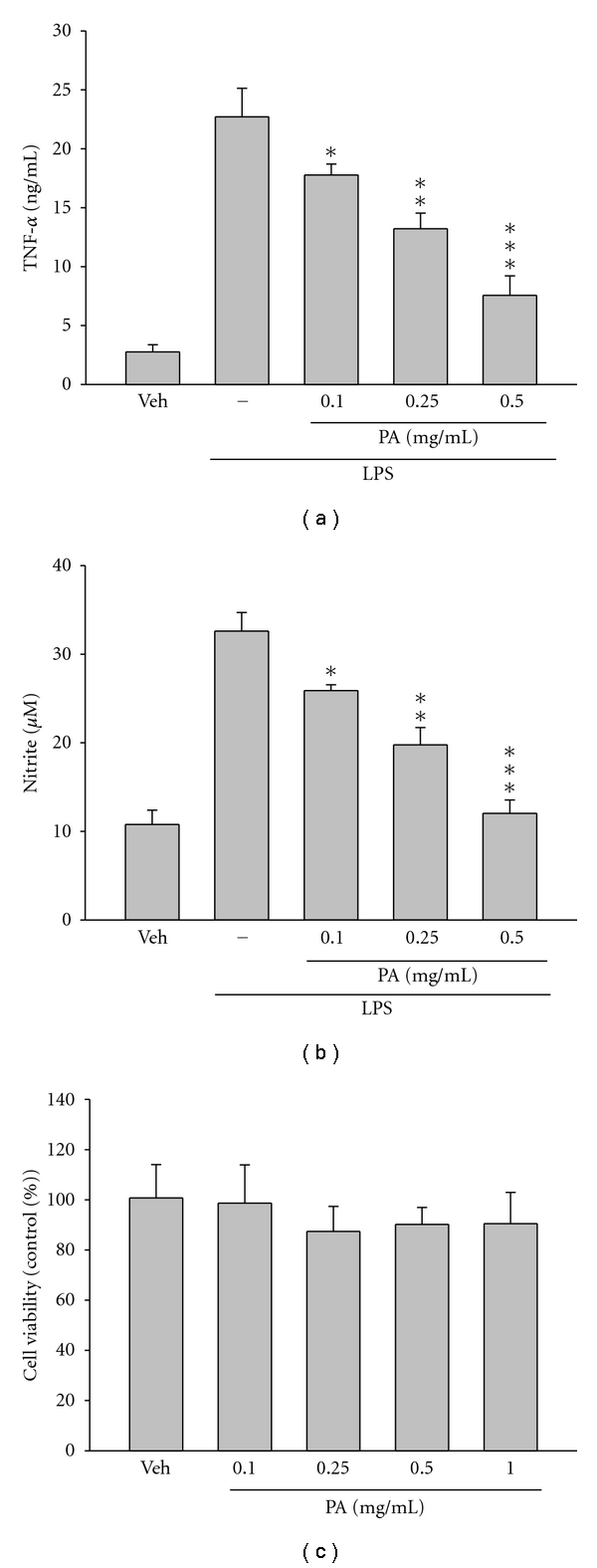
PA-inhibited LPS-induced proinflammatory cytokine and mediator productions. RAW 264.7 cells were pretreated with PA (0.1–0.5 mg/mL) for 30 min, and then stimulated with LPS (100 ng/mL). Culture media were collected at 24 h for (a) TNF-*α* and (b) nitrite analysis. (c) Cell viability in PA-treated cells was evaluated using the MTT assay. The results are displayed in percentage of control samples. Data are presented as mean ± SEM (*n* = 3) for three independent experiments; *P < 0.05, **P < 0.01, ***P < 0.001 as compared with the LPS treatment.

Proinflammatory cytokines and mediators play important roles in the inflammatory process. To further validate the effect of PA on antiinflammatory function *in vitro*, the levels of secreted TNF-*α* and NO were measured in mouse peripheral macrophage RAW 264.7 cells when treated with LPS. As demonstrated in Figure 6, treatment of RAW 264.7 cells with LPS (100 ng/mL) caused a substantial increase in the production of TNF-*α* and NO. However, pretreatment with PA before being incubated with LPS resulted in a dose-dependent inhibition of the LPS-induced TNF-*α* and NO production in RAW 264.7 cells (Figures 6(a) and 6(b)). To examine whether PA is cytotoxic to the cells, RAW 264.7 cells were incubated with 0.1–1.0 mg/mL of PA for 24 h. Within our tested concentrations, no cytotoxic effect of PA was observed (Figure 6(c)).

### 3.6. The Antiinflammatory Effects of PA Are via Downregulating the Protein Levels of iNOS and COX-2

**Figure 7:**
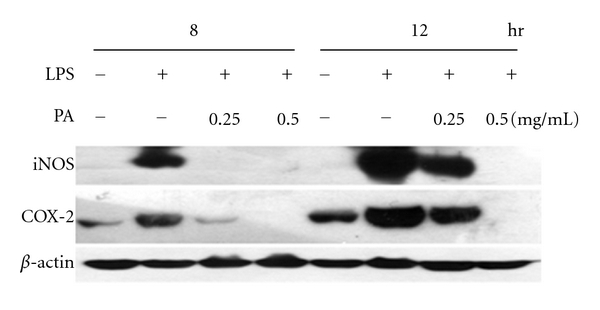
PA decreased the protein levels of iNOS and COX-2 in LPS-stimulated macrophages. RAW 264.7 cells were pretreated with PA (0.25–0.5 mg/mL) for 30 min, and then stimulated with LPS (100 ng/mL) for 8–12 h. *β*-actin was used as an internal loading control.

To determine if the inhibitory effect of PA on these inflammatory mediators was related to the regulation of iNOS and COX-2, the levels of these two proteins were examined by Western blot analysis at 8 and 12 h after LPS treatment. As shown in Figure 7, the protein levels of iNOS and COX-2 were markedly increased upon LPS treatment, and these inductions were drastically blocked by treatment with PA (0.5 mg/mL).

### 3.7. Prevention of LPS-Induced NF-*κ*B Activation by PA

**Figure 8:**
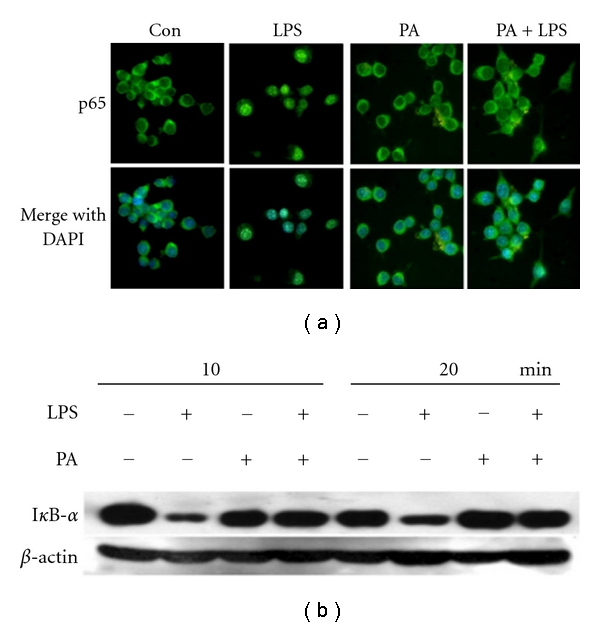
Prevention of LPS-induced NF-*κ*B activation by PA. (a) PA inhibits LPS-induced nuclear translocation of NF-*κ*B p65 subunit. The subcellular localization of NF-*κ*B p65 subunit was detected by immunofluorescence with an antibody especially against p65 as described in Section 2. The same fields were counterstained with DAPI for location of nuclei. (b) PA blocks LPS-induced I*κ*B*α* degradation. Protein extracts were separated by SDS-PAGE followed by Western blot analyses antibody especially against I*κ*B*α*. *β*-actin was used as an internal loading control.

NF-*κ*B is an important transcriptional regulator of inflammatory cytokines, and it plays a crucial role in immune responses [23]. To determine if PA would inhibit the expression of the proinflammatory mediators through the suppression of NF-*κ*B activation, we examined the regulatory effect of PA on LPS-induced nuclear translocation of the cytosolic NF-*κ*B p65 subunit by immunostaining. As expected, treatment of LPS-stimulated nuclear translocation of p65 (Figure 8(a), LPS). However, treatment of PA markedly suppressed the LPS-induced NF-*κ*B p65 nuclear translocation (Figure 8(a), PA + LPS). Since nuclear translocation of NF-*κ*B was preceded by the degradation of I*κ*B, we next examined the effect of PA on LPS-induced I*κ*B degradation by Western blot analyses. As demonstrated in Figure 8(b), the stimulation of RAW 264.7 macrophages with 100 ng/mL LPS induced a rapid degradation of cytosolic I*κ*B protein within 10 to 20 min; this effect was drastically blocked by the treatment with PA (0.5 mg/mL).

## 4. Discussion

In Taiwan, *Plectranthus amboinicus* (Lour.) Spreng. (PA) is a common folk medicine for summer cold, scald, wounds, and bites from bugs or mosquitoes. However, the scientific theories behind these therapeutic effects are still unclear. Here we report new insights into the functions and possible mechanisms of PA, including (1) its analgesic ability demonstrated by two different analgesic test methods: acetic acid-induced writhing response and formalin test, (2) its antiinflammatory ability demonstrated by decreasing the swelling of carrageenan-induced mice paw edema and the levels of proinflammatory mediators (TNF-*α* and COX-2), (3) its antioxidant ability demonstrated by increased SOD and GRx levels and decreased MDA level, (Notably, PA does not increase the level of GPx like the indomethacin does), and (4) possible mechanisms of its antiinflammatory activities.

The analgesic ability of PA was evaluated using two different animal models. Intraperitoneal injection of acetic acid causes an increase of prostaglandins in peritoneal fluids such as PGE_2_ and PGF_2*α*_, serotonin, and histamine involved in part, which was a model commonly used for screening peripheral analgesics [24]. The formalin test is a tonic model of continuous pain resulting from formalin-induced tissue injury. It is a widely used model, particularly for the screening of novel compounds, since it encompasses inflammatory, neurogenic, and central mechanisms of nociception [25]. The results showed that the PA considerably inhibited acetic acid-induced writhing in mice (Figure 2) and the late-phase pain response, not the neurogenic (early-phase) pain, caused by intraplantar injection of formalin (Figure 3). Such results suggested that *Plectranthus amboinicus* (Lour.) Spreng. possessed remarked analgesic activity. 

Carrageenan-induced paw edema in mice has been accepted as a useful phlogistic tool for investigating antiinflammatory agents. There are biphasic effects in carrageenan-induced edema [22]. The early hyperemia results from the release of histamine and serotonin and the delayed phase of carrageenan-induced edema results mainly from the potentiating effects of bradykinin on mediator release, and of prostaglandins producing edema after the mobilization of leukocytes. According to Figure 4(a), PA showed effectively inhibitory activity on carrageenan-induced paw inflammation over a period of 4 h at the dose of 1.0 g/kg, which was comparable to that of indomethacin, which indicated its action against neutrophils migration and release of histamine, serotonin, and kinins in early phase, and prostaglandin in later phase. Furthermore, considering the crucial role of COX-2 expression and cytokines production in the progress of inflammation in injury area, COX-2 and TNF-*α* contents were also examined in this study. COX-2 is an inducible enzyme found in activated inflammatory cells that creates prostanoid mediators. Inhibition of COX-2 protein expression has also become the most popular and valid method for studying antiinflammatory effects both in *in vivo* and *in vitro* models [8]. TNF-*α*, a key mediator in inflammatory response, stimulates innate immune responses by activating T cells and macrophages that stimulate the release of other inflammatory cytokines. TNF-*α* is also a mediator of carrageenan-induced inflammation and is able to enhance the further release of kinins and leukotrienes, which is suggested to have an important role in the maintenance of long-lasting nociceptive response [26]. The production of TNF-*α* in edema paw tissues induced by carrageenan was decreased by PA treatment (Figure 4(c)). PA also significantly restrained the protein expression of COX-2 in the edema paw tissues of mice (Figure 4(d)). Therefore, such results revealed that *Plectranthus amboinicus* (Lour.) Spreng. displayed significantly antiinflammatory activities in the model of carrageenan-induced paw edema of mice, *via* inhibiting vascular permeability, which might be related to the reduction of COX-2 and TNF-*α*.

The carrageenan-induced inflammatory response has been linked to the neutrophil infiltration, the release of neutrophil-derived mediators, as well as the production of neutrophil-derived free radicals, such as hydrogen peroxide, superoxide, and hydroxyl radicals [27], and the production of MDA is due to the attack of plasma membranes by free radicals [28, 29]. Previous studies consider that endogenous glutathione plays an important role against carrageenan-induced local inflammation [30]. Glutathione is a known oxyradical scavenger and the enhancement of glutathione levels favor reducing MDA level [31]. In this study, significantly increase in SOD and GRx activities with PA treatment was found (Figure 5); contemporaneously, there was a significant decrease in MDA level with PA treatment (Figure 4(b)). We assume that the suppression of MDA production is probably due to the increase of SOD and GRx activities. The safety study showed that PA (10 g/kg, p.o.) did not produce any death or behavioral changes in the treated mice (data not shown).

Finally, LPS-stimulated NO and TNF-*α* release from RAW 264.7 macrophages was used to evaluate the mechanism of the aqueous extract of PA *in vitro*. As shown in Figure 6, PA potently and dose dependently inhibited the elevation of TNF-*α* and NO level induced by LPS in macrophages which further proved the antiinflammatory activities of PA. Furthermore, we examined the levels of iNOS and COX-2, as shown in Figure 7, the protein levels of iNOS and COX-2 induced by LPS were drastically blocked by pre-treatment with PA. NF-*κ*B is known to be a major transcription factor to regulate the expressions of proinflammatory enzymes and cytokines, such as iNOS, COX-2, and TNF-*α* [7]. NF-*κ*B subunits (p65 and/or p50) are normally sequestered in the cytosol as an inactive complex by binding to its inhibitory factor, I*κ*B-*α*, in unstimulated cells. Upon the stimulation of proinflammatory signals including LPS, I*κ*B-*α* is phosphorylated by I*κ*B-*α* kinase (IKK) and inactivated through ubiquitin-mediated degradation. The resulting free NF-*κ*B is translocated into the nucleus and acts as a transcription factor. As shown in Figure 8, the treatment with PA effectively blocks the degradation of I*κ*B and activation of NF-*κ*B in RAW 264.7 macrophages stimulated by LPS. Therefore, these results suggest that PA inhibits the expression of iNOS and COX-2, and thus NO production through inactivation of NF-*κ*B by reducing I*κ*B-*α* degradation. These *in vitro *findings were well correlated with the *in vivo* antiinflammatory effects of PA. 

Inhibition of inflammatory cytokine and mediator production or function serves as a key mechanism in the control of inflammation, and agents that suppress the expression of these inflammation-associated genes have therapeutic potential in the treatment of inflammatory diseases. However, the most common used nonsteroidal antiinflammatory drugs can cause gastric erosions, exacerbate asthma, and cause kidney and liver damages. Therefore, natural products have attracted interest as potential therapeutic agents for the treatment of inflammation. 

Separation and determination of active chemical constituents are generally recommended for standardization and quality control of herbal products. Moreover, the identification of the major compound in an herb or herbal extract could elucidate pharmacological activity of the herbal extract. The *Plectranthus amboinicus *(Lour.) Spreng (Lamiaceae) is a native species in Asia. Previous studies reported that the leaves contain phytocompounds as essential oils, flavonoids, terpenes, and cinnamic derivates which possess antiinflammatory and chemopreventive effects [32, 33]. Our findings revealed that the aqueous extracts from *P. amboinicus* posses a promising analgesic and antiinflammatory activities. These effects were mediated by inhibiting the proinflammatory mediators through blocking NF-*κ*B activation. Phytochemical analysis shows that the aqueous extracts of *P. amboinicus* contain plentiful carvacrol. Carvacrol has been demonstrated to possess significant antiinflammatory activities in previous studies [34, 35]. Therefore, it should be reasonable to suggest that analgesic and antiinflammatory profile of *P. amboinicus* might be related to the presence of the phytocompounds, especially the carvacrol. Meanwhile, these effects observed in this study provide evidence for folkloric uses of PA in relieving pain and inflammation. 

In this study, PA clearly showed its analgesic and antiinflammatory activities. The mechanisms by which PA exerts its analgesic as well as antiinflammatory effect are correlated with the inhibition of iNOS and COX-2 expression via inactivation of NF-*κ*B, and this serves as a possible rationale for the use of *Plectranthus amboinicus* (Lour.) Spreng. in traditional medicine for antiinflammation.
